# 云南宣威、富源地区非吸烟女性肺癌生存分析

**DOI:** 10.3779/j.issn.1009-3419.2019.08.01

**Published:** 2019-08-20

**Authors:** 继华 李, 俊 何, 云生 张, 云超 黄, 石安 刘, 云 李, 军 许, 兴舟 何, LAN Qing

**Affiliations:** 1 655000 曲靖，曲靖市疾病预防控制中心 Qujing Centers for Disease Control and Prevention, Qujing 655000, China; 2 650118 昆明，云南省肿瘤医院 Yunnan Cancer Hospital, Kunming 650118, China; 3 香港大学医学院公共卫生学院 School of Public Health, Li Ka Shing Faculty of Medicine, The University of Hong Kong, Hong Kong, China; 4 100050 北京，中国疾病预防控制中心 Chinese Centers for Disease Control and Prevention, Beijing 100050, China; 5 20892 Bethesda, National Cancer Institute, MD, USA National Cancer Institute, National Institutes of Health, Bethesda 20892, MD, USA

**Keywords:** 肺肿瘤, 生存分析, 预后因素, 妇女, 宣威, 富源, Lung neoplasms, Survival, Female never-smokers, Prognostic factors, Xuanwei, Fuyuan

## Abstract

**背景与目的:**

云南东部农村地区宣威市、富源县女性居民主要从事农业生产和家务工作，基本不吸烟，但肺癌死亡率却是世界上最高的，而且发病、死亡年龄提前。本研究对宣威、富源非吸烟女性肺癌生存状况及其影响因素进行分析。

**方法:**

以2006年-2010年被当地省、市、县9家医院新诊断、并纳入“非吸烟女性肺癌病例对照研究项目”的常住户籍女性肺癌病例为研究对象随访至2016年末。通过Life-table法进行全部病例生存分析，评估人群相对生存率和年龄别标化相对生存率。应用*Kaplan-Meier*法和*Cox*比例风险模型分别进行单因素生存分析、分层分析和多因素分析。

**结果:**

随访的1, 250例病例中，死亡1, 075例，删失175例，随访中位时间为69个月(95%CI: 61.9-76.0)。病例平均年龄(54.8±10.9)岁，Ⅰ期、Ⅱ期、Ⅲ期、Ⅳ期和未知分期分别占3.5%、8.7%、20.7%、29.7%和37.4%；手术、非手术治疗和未治疗分别占17.2%、39.0%和43.8%，组织学、细胞学诊断占51.6%。中位生存时间13.2个月，5年观察生存率、相对生存率、年龄标化相对生存率分别为8.9%(95%CI: 7.0-10.6)、9.4%(95%CI: 7.6-11.5)和10.1%(95%CI: 3.7-20.5)。Ⅰ期、Ⅱ期、Ⅲ期、Ⅳ期、未分期5年生存率分别为41.1%、22.4%、5.3%、1.3%、11.2%；手术治疗、非手术治疗、未治疗分别为34.8%和3.2%、4.7%；腺癌、鳞癌分别为17.9%和5.6%。省级医院治疗、X线胸部筛查、非农民职业、城镇居住、65岁以下年龄等因素有利于提高生存率，而市县级医院治疗、农民职业、乡村居住、65岁以上年龄等则生存率较低。分层分析显示，任意原发灶-淋巴结-远处转移(tumor-node-metastasis, TNM)分期，无论腺癌或鳞癌患者，行手术治疗的生存率明显高于非手术治疗；与未治疗病例相比非手术治疗仅在Ⅲ期显示差异；腺癌生存率大于鳞癌不仅仅因为早期和手术病例较多，在Ⅲ期、未分期也显示明显生存优势。不同级别医院治疗疗效有明显差异，省级医院治疗的Ⅳ期、鳞癌的生存预后明显优于市、县级医院。*Cox*分析显示治疗方法、TNM分期、治疗医院级别、X线胸部筛查是独立预后因素，其中TNM分期、手术治疗对肺癌患者生存影响较大，而治疗医院级别、X胸部筛查相对较弱。

**结论:**

宣威、富源非吸烟女性肺癌生存率较低，主要与其诊断时早期病例和手术、综合治疗较少、而未治疗病例较多有关，其次较差的农村社会经济、健康保障等也是生存预后的不利因素。

## Abstract (en)

**Background and objective:**

Lung cancer rates among women in rural Xuanwei and Fuyuan counties in eastern Yunnan province, China, are among the highest in the world, even though almost all women are non-smokers, and they tend to develop lung cancer at a younger age than other locations by roughly 5 yr-10 yr. This study investigated the survival of lung cancer patients among female never-smokers.

**Methods:**

The female never-smokers, who had lived for many years in Xuanwei and Fuyuan counties, with lung cancer newly-diagnosed between July 2006 to March 2010, were followed up through the end of 2016. Age-standardized relative survival for all cases was calculated using local life table. The *Kaplan-Meier* method and *Log-rank* test were used to analyze the relationship between the variables and the prognosis in univariate analysis. *Cox* regression analysis was employed in the multivariate analysis.

**Results:**

Among 1, 250 total subjects, 1, 075 died and the remaining 175 were censored during the follow-up, with a median follow-up period of 69 months (95%CI: 61.9-76.0). Overall, the mean age was (54.8 ±10.9) yr, with variable clinical stages: 3.5% of cases were stage Ⅰ, 8.7% stage Ⅱ, 20.7% stage Ⅲ, 29.7% stage Ⅳ, and 37.4% stage unknown. The 645 patients(51.6%) with cyto-histological diagnosis contains 303 with Squamous cell carcinoma, and 231 with adenocarcinoma, 24 with small cell, 43 with other specified type and 39 with unknown histological type. Only 215 (17.2%) patients received surgery, 487 (39.0%) were treated without surgery, and 548 (43.8%) did not receive any specific anticancer therapy. The 5-year observed survival rate and age-standardized relative survival were 8.9% (95%CI: 7.0-10.6), and 10.1% (95%CI: 3.7-20.5) respectively, with a median survival of 13.2 months. The 5-year survival rates were 41.1% for stage Ⅰ, 22.4% for stage Ⅱ, 5.3% for stage Ⅲ, 1.3% for stage Ⅳ, 11.2% for missing stage, 17.9% for adenocarcinoma, and 5.6% for squamous cell carcinoma respectively. Surgery significantly improved 5-year survival rate compared with non-surgery (34.8% *vs* 3.2%, *P* < 0.001). The patients with non-treatment, aged 65 years and older, living in rural areas and farmer with low socioeconomic status had poorer survival, whereas the patients treated in provincial hospitals and chest X-ray screening had better survival. *Cox* multivariate analysis further showed that stage of tumor-node-metastasis (TNM), treatment status, hospital-level, and X-ray screening were factors correlated with survival.

**Conclusion:**

Patients with lung cancer among female never-smokers in Xuanwei and Fuyuan experience poorer survival, because they are less likely to be diagnosed at early stage, as well as less likely to receive surgery and comprehensive treatment. Furthermore low socioeconomic status and poor health security are also responsible for the low survival.

云南东部宣威市、富源县等农村地区女性基本不吸烟，而肺癌疾患却是世界上最严重的地区，自20世纪70年代以来多次调查均显示当地的肺癌发病率、死亡率较高^[1-4]^，肺癌死亡占全部恶性肿瘤的50%左右，而且发病、死亡年龄较其他地区提前5岁-10岁^[1, 4]^，对当地居民生命健康以及经济发展造成较大的影响。本研究选择宣威、富源非吸烟女性肺癌病例进行随访，观察分析生存状况及其影响因素。

## 1 材料与方法

### 1.1 病例资料

病例来源为2006年7月-2010年3月经县、市、省级9家医院(各3家)通过计算机断层扫描(computed tomography, CT)、细胞学、病理学新诊断的宣威市、富源县常住户籍的女性肺癌病例(美国国家肿瘤研究所合作项目“宣威、富源非吸烟女性肺癌病例对照研究”研究对象)。为促使当地的肺癌病例最大可能地被纳入研究和保证诊断质量，研究项目给予X线胸片检查发现的疑似病例进行胸部CT、多批次痰样薄层液基细胞学涂片检查和2个月后CT复查等免费措施，相关蜡块、切片、细胞涂片、CT等影像资料经中国医学科学院肿瘤医院复核，并依据国际抗癌联盟(Union for International Cancer Control, UICC)第七版国际肺癌原发灶-淋巴结-远处转移(tumor-node-metastasis, TNM)分期准则^[5]^对病例进行TNM分期。共征集1309例非吸烟女性肺癌病例(其中100例肺癌为2007年滇东农民肺癌流行病学调查X线胸片筛查发现^[6]^)，占当地同期新诊断病例(1, 495例)的87.6%。

### 1.2 随访

采用主动与被动相结合的方法进行初、中、后三期随访。初期随访于病例征集后1个月、2个月、12个月、24个月，由通过培训的诊治医院调查员进行电话随访，及时与病人及其家属联系，了解病情转归、新近诊疗信息，并指导病人按时胸部CT、痰检复查。中期随访(2011年-2013年)于医院全部病例征集完成后，由专门随访小组对全部病例进行入户随访。后期随访(2014年-2016年)通过查询死因监测和医疗保险系统、医院入院诊疗记录，跟踪早、中期随访存活病例的相关信息，并辅以电话随访。

### 1.3 统计分析

采用SPSS 19.0和EXCEL 2016进行统计分析。应用Life-table法计算全部肺癌患者的观察生存率(observed survival, OS)、中位生存时间; 根据2000年、2010年当地女性人口完全寿命表为依据，采用Ederer Ⅱ法^[7, 8, 9]^推算当地期望生存率(${\rm{S}}_{\rm{i}}^* $)，计算人群肺癌相对生存率(relative survival, RS)及其标准误^[9, 10]^;同时以世界肺癌标准年龄人口^[11]^计算年龄标化相对生存率(age standardized relative survival, ASRS)^[9, 10]^。应用*Kaplan-Meier*法及分层分析法^[12]^(显著性检验为*Log-rank*法，检验水准*α*=0.05)对临床变量、社会环境因素不同水平的生存曲线进行比较，并用*Log-rank*法计算单变量危险比(hazard ratio, HR)^[13-16]^，评估影响因素的生存效应。

$
\mathrm{HR}=\frac{\mathrm{O}_{\mathrm{a}} / \mathrm{E}_{\mathrm{a}}}{\mathrm{O}_{\mathrm{h}} / \mathrm{E}_{\mathrm{h}}}
$

式中O_a_、O_b_分别为*Kaplan-Meier*法计算的某一随访期间的第k组(a组或b组)观察到的事件(死亡)数O_k_，E_a_、E_b_指假设存在的无生存差异的零假设的相应组预期事件(死亡)数E_k_。

$
			 {\rm{O}}_{\mathrm{k}}=\sum\limits_{\mathrm{i}=1}^{\mathrm{i}} \mathrm{d}_{\mathrm{ki}}
			  $

$
			  \mathrm{E}_{\mathrm{k}}=\sum\limits_{\mathrm{i}=1}^{\mathrm{i}} \frac{\mathrm{D}_{\mathrm{i}}}{\mathrm{N}_{\mathrm{i}}} \mathrm{n}_{\mathrm{ki}}
			  $

式中N_i_、D_i_为在生存时间(t_i_)比较的两组或多组合计起初观察人数和死亡人数; n_ki_为k组(a组或b组)在生存时间(t_i_)起初观察人数。

危险比的95%CI：

$
			  \text{EXP}((\ln (\mathrm{HR}) \pm 1.96 \times \sqrt{\left(1 / \mathrm{E}_{\mathrm{a}}+1 / \mathrm{E}_{\mathrm{b}}\right)})
			  $

对单因素生存分析有意义的变量，且TNM分期、组织学资料完整的病例进行*Cox*比例风险模型多因素分析，筛选出对生存具有独立预测作用的因素，并用*Log minus log*图(生存函数的二重对数曲线)验证*Cox*比例风险假设。

## 2 结果

### 2.1 病例特征

截止2016年12月31日，随访排除肺癌嫌疑13例(0.99%)，因病例联系电话、地址有误或搬迁或其他原因未能随访46例(3.5%)，完成随访1, 250例(95.5%); 中位随访时间69个月(95%CI: 61.9-76.0)(reverse *K-M*法计算^[17]^)，随访病例死亡1, 078例(86.2%)，截尾数据172例(13.8%)。病例背景、临床特点见表 1，诊断时的平均年龄(54.8±10.9)岁。病理学、细胞学诊断645例(医院常规诊断和专门设置的薄层液基细胞学痰样实验室诊断)，占全部病例的51.6%，其中腺癌、鳞癌、小细胞癌分别占38.8%、47.6%和3.7%(医院手术、气管镜、淋巴结穿刺、经皮肺穿刺活检、痰检等确诊的410例中，腺癌、鳞癌、小细胞分别占52.1%、24.6%、4.42%)。进行临床TNM分期783例，占62.6%;未分期467例，占37.4%(主要是搜集的信息不足)。手术治疗215例(17.2%)，非手术住院治疗487例(39.0%)，门诊对症治疗或未治疗(简称未治疗)548例(43.8%)。不同治疗措施间的年龄、职业、城乡、临床分期、组织学类型、治疗医院级别构成不完全一致(*P* < 0.05)。随访病例经组织学诊断比例不高，但样本量较大(占当地同期新诊断肺癌的87.6%)、病例来源广(既有医院治疗病例，也包括大量的未治疗病例)，同时随访结果显示未经组织学诊断病例中位生存时间、5年生存率明显小于组织学诊断病例(表 2)，病例诊断质量相对可靠。

**1:** 宣威、富源县非吸烟女性肺癌病例人口学和临床特征(2006年-2010年) Demographic and clinical characteristics of non-smoking Women with lung cancer in Xuanwei and Fuyuan county diagnosed in 2006-2010

Characteristics	All			Surgery			Inpatient without surgery			Untreated		*χ*^2^	*P*
	No.	%		No.	%		No.	%		No.	%		
Number of patients	1, 250			215	100.0		487	100.0		548	100.0		
Age (yr)												67.18	0.000
25-44	246	19.7		71	33.0		101	20.7		74	13.5		
45-54	359	28.7		72	33.5		152	31.2		135	24.6		
55-64	375	30.0		49	22.8		136	27.9		190	34.7		
≥65	270	21.6		23	10.7		98	20.1		149	27.2		
Occupation												27.65	0.000
Non-farmer	83	6.6		19	8.8		47	9.7		17	3.1		
Farmer	1, 167	93.4		196	91.2		440	90.3		531	96.9		
Address												19.86	0.000
Urban	254	20.3		64	29.8		113	23.2		77	14.1		
Rural	996	79.7		151	70.2		374	76.8		471	85.9		
Hospital level of service												311.37	0.000
Province	278	39.6		114	53.0		164	33.7					
City and county	424	60.4		101	47.0		323	66.3					
Screening with chest X												4.92	0.085
Unscreened	1, 150	92.0		190	88.4		454	93.2		506	92.3		
Screened	100	8.0		25	11.6		33	6.8		42	7.7		
Histological type												554.35	0.000
Adenocarcinoma	231	18.5		137	63.7		81	16.6		13	2.4		
Squamous cell	307	24.6		31	14.4		110	22.6		166	30.3		
Small cell	24	1.9		6	2.8		13	2.6		5	0.9		
Unknown^*^	39	3.1		39	18.1		0	0.0		0	0.0		
Other specified types	44	3.5		2	0.9		39	8.0		3	0.5		
Missing pathology diagnosis	605	48.4		0	0.0		244	50.1		361	65.9		
Clinical stage												81.99	0.000
Ⅰ	44	3.5		20	9.3		10	2.1		14	2.6		
Ⅱ	109	8.7		42	19.5		28	5.7		39	7.1		
Ⅲ	259	20.7		33	15.3		96	19.7		130	23.7		
Ⅳ	371	29.7		38	17.7		173	35.5		160	29.2		
Missing/Unknown	467	37.4		82	38.1		180	37		205	37.4		
^*^The cases with surgery cannot find histological type information.													

**2:** 不同年龄组、TNM分期、组织学、治疗方式及职业、居住位置的女性非吸烟肺癌患者生存状况 Lung cancer survival in non-smoking women in Xuanwei and Fuyuan counties, by age, socio-economic status, TNM stage, histology and treatment status

Index	*n*	Medians survival time (mon, 95%CI)	5-year survival (%, 95%CI)	Hazard ratio (95%CI)	*P*
Age (yr)					
25-44	246	13.0 (10.6-15.4)	9.8 (5.8-15.1)	1.00 (ref)	
45-55	359	13.0 (11.6-14.4)	10.8 (7.4-14.8)	1.06 (0.89-1.26)	0.506
55-64	375	13.0 (11.0-15.0)	9.1 (6.1-12.7)	1.07 (0.90-1.27)	0.476
≥65	270	11.0 (9.1-12.9)	7.1 (4.2-11.0)	1.20 (1.00-1.45)	0.048
Residence areas					
Urban	83	14.0 (11.9-16.1)	12.1 (7.9-17.2)	1.00 (ref)	
Rural	1, 167	12.0 (10.8-13.2)	8.6 (6.7-10.8)	1.21 (1.05-1.39)	0.012
Occupation					
Non-farmer	254	16.0 (12.7-19.3)	17.9 (9.5-28.3)	1.00 (ref)	
Farmer	996	12.0 (10.9-13.1)	8.7 (6.9-10.7)	1.42 (1.05-1.39)	0.005
Hospital level of service					
Province	278	19.0 (16.6-21.4)	18.1 (13.5-23.3)	1.00 (ref)	
Municipal and county	424	11.0 (10.0-12.0)	8.5 (5.5-12.4)	1.57 (1.38-1.79)	0.000
X-ray screen					
Screened	1150	15.0 (9.6-20.4)	15.3 (8.4-24.2)	1.00 (ref)	
Unscreened	100	12.0 (10.9-13.1)	8.7 (7.0-10.8)	1.29 (1.06-1.58)	0.020
Identified with cytology and histology			
Yes	645	14.0 (12.0-15.4)	12.2 (9.5-15.3)	1.00 (ref)	
No	605	11.0 (9.7-12.3)	6.3 (4.3-8.7)	1.33 (1.18-1.50)	0.000
Pathological type					
Adenocarcinoma	231	22.0 (16.6-27.4)	17.9 (12.5-24.2)	1.00 (ref)	
Squamous	303	11.0 (9.4-12.6)	5.6 (3.2-9.0)	1.77 (1.49-2.11)	0.000
Small cell	24	11.0 (9.4-12.6)	NPE^*^	1.98 (1.19-3.30)	0.001
Unknown	39	32.0 (9.3-54.7)	28.9 (13.9-45.7)	0.75 (0.57-0.98)	0.114
Other specified types	44	16.0 (11.4-20.6)	10.5 (2.7-24.7)	1.14 (0.83-1.56)	0.436
Clinical stage					
Ⅰ	44	57.0 (47.7-66.3)	41.1 (23.0-58.5)	1.00 (ref)	
Ⅱ	109	33.0 (26.2-39.8)	22.4 (14.0-32.1)	1.68 (1.28-2.20)	0.008
Ⅲ	259	13.0 (10.9-15.1)	5.3 (2.8-9.1)	3.39 (2.61-4.39)	0.000
Ⅳ	371	7.0 (5.8-8.2)	1.3 (0.4-3.5)	5.40 (4.15-7.02)	0.000
Missing stage	467	14.0 (11.9-16.1)	11.2 (8.3-14.7)	2.87 (2.25-3.65)	0.000
Treatment status					
Surgery	215	45.0 (36.1-53.9)	34.8 (27.7-42.0)	1.00 (ref)	
Treatment without surgery	487	11.0 (9.7-12.3)	3.2 (1.7-5.5)	2.78 (2.39-3.23)	0.000
Non-treated	548	10.0 (8.7-11.3)	4.7 (3.0-7.0)	2.78 (2.41-3.22)	0.000
^*^NPE: not possible to estimate. TNM: tumor-node-metastasis.					

### 2.2 生存概况

全部随访病例中位生存时间13.2个月，1年、3年、5年、8年OS、RS、ASRS见表 3。

**3:** 宣威、富源县非吸烟女性肺癌生存率、相对生存率和年龄标化相对生存率(2006年-2010年诊断) Observed survival, relative survival and age-standardized relative survival rates for patients with lung cancer in non-smoking women in Xuanwei and Fuyuan counties, diagnosed in 2006-2010

Time since diagnosis	Observed survival (%, 95%CI)	Relative survival (%, 95%CI)	Age-standardized relative survival (%, 95%CI)
1-year	45.0 (42.2-47.8)	45.6 (42.7-48.5)	43.7 (40.9-46.4)
3-years	18.6 (16.4-21.0)	19.4 (17.1-21.9)	17.9 (11.7-25.2)
5-years	8.9 (7.0-10.6)	9.4 (7.6-11.5)	10.1 (3.7-20.5)
8-years	1.4 (0.7-2.7)	1.7 (0.8-3.1)	1.4 (0.0-99.9)

### 2.3 单因素生存分析

表 2所示，从低年龄组至高年龄组生存状况呈逐渐恶化的趋势，但仅有65岁以上与45岁前有统计学差异(HR=1.20, *P*=0.048)。城镇居民Ⅰ期、Ⅱ期病例、手术治疗、住院治疗所占比例高于乡村(Ⅰ期、Ⅱ期：14.6% *vs* 11.6%，*P* < 0.05;手术治疗：25.2% *vs* 15.2%，*P* < 0.001;住院治疗：44.5% *vs* 37.5%，*P* < 0.001)，其生存状况优于乡村居民(*P* < 0.05)。非农民职业病例占6.6%，Ⅰ期、Ⅱ期病例和手术治疗比例与农民相近，但其住院治疗比例、尤其是省级医院比例却明显较高(住院治疗：79.5% *vs* 54.5%，*P* < 0.001;省级医院治疗：53.6% *vs* 19.8%，*P* < 0.001)，其生存状况优于农民(*P* < 0.01)。X线胸片筛查病例中Ⅰ期、Ⅱ期所占比例与未筛查者一致(12.0% *vs* 12.2%, *P* > 0.05)，但接受手术治疗比例高于后者(25.0% *vs* 16.0%，*P*=0.085)，其生存率也略高于未经筛查者(*P* < 0.05)。

细胞学、组织学诊断病例与仅有CT和临床诊断病例的TNM分期构成无明显差异(Ⅰ期、Ⅱ期：12.4% *vs* 12.1%; Ⅲ期：22.5% *vs* 18.8%; Ⅳ期：31.9% *vs* 27.6%; *P* > 0.05)，但手术治疗比例明显较多(33.3% *vs* 0.0%, *P* < 0.001)，而未治疗病例较少(29.0% *vs* 59.7%, *P* < 0.001)，其生存预后明显优于仅有CT、临床诊断的病例(*P* < 0.001)。腺癌病例的Ⅰ期、Ⅱ期和手术治疗比例明显高于其他组织类型(Ⅰ期、Ⅱ期：21.6% *vs* 10.1%，*P* < 0.001;手术：59.3% *vs* 7.6%，*P* < 0.001)，生存预后优于鳞癌、小细胞癌，而与其他组织类型和不详组织类型(手术切除病例，其组织类型不详)一致(*P* > 0.05)。鳞癌与小细胞癌的生存率差异不大(*P* > 0.05)。

省级医院治疗的病例中手术比例高于市级、县级医院(手术：41.0% *vs* 23.8%，*P* < 0.001)，而且Ⅳ期、鳞癌病例较少(Ⅳ期：36.4% *vs* 53.2%，*P* < 0.01;鳞癌：16.8% *vs* 44.4%，*P* < 0.001)，其生存预后优于市级、县级医院(*P* < 0.001)。

各TNM临床分期间的生存率差距较大，从Ⅰ期-Ⅳ期依次减小(线性趋势*P* < 0.001)。手术治疗病例的Ⅰ期、Ⅱ期所占比例高于非手术者(28.8% *vs* 8.8%, *P* < 0.001)，其5年生存率是后者的8.7倍(34.8% *vs* 3.2%, *P* < 0.001); 非手术治疗住院患者的Ⅳ期肺癌比例高于未治疗者(35.5% *vs* 29.2%, *P* < 0.05)，二者生存率差异不大(*P* > 0.05)。

### 2.4 分层分析

按治疗方法、TNM分期、组织类型分层分析显示(表 4)：各分期和腺癌、鳞癌的省级医院治疗生存预后优于市、县级医院，但仅在Ⅳ期、鳞癌存在统计学意义，省级医院的Ⅳ期、鳞癌死亡危险性分别降低了35.6%和54.6%;不同级别医院的手术或非手术治疗的生存状况差异没有统计学意义(*P* > 0.05)。

**4:** TNM分期、组织学类型、治疗方式分层的省级与市县级医院治疗病例生存状况对比 Stratified analyses of hospital-level on survival by treatment status, stage, histologic cell type

Index	Provincial hospitals			Municipal and county hospitals		Hazard ratio^*^ (95%CI)	*P*
	*n*	Median survival (mon, 95%CI)		*n*	Median survival (mon, 95%CI)		
TNM stage							
Ⅰ	8	70 (51.8-88.2)		22	57 (45.4-68.6)	2.10 (0.71-6.25)	0.217
Ⅱ	29	56 (41.9-70.1)		41	33 (27.6-38.4)	1.51 (0.87-2.64)	0.115
Ⅲ	50	18 (14.8-21.2)		79	14 (11.3-16.7)	1.14 (0.78-1.66)	0.478
Ⅳ	50	11 (7.2-14.8)		161	6 (4.3-7.7)	1.55 (1.15-2.10)	0.005
Missing/Unknown	141	19 (16.1-21.9)		121	12 (7.9-16.1)	1.27 (0.97-1.66)	0.068
Histological subtype							
Adenocarcinoma	114	22 (15.9-28.1)		104	26 (13.3-38.7)	0.88 (0.65-1.20)	0.664
Squamous cell	38	22 (12.3-31.7)		103	10 (7.4-12.6)	2.20 (1.55-3.13)	0.000
Treatment status							
Surgery	114	50 (41.9-58.1)		101	34 (25.6-42.4)	1.05 (0.75-1.48)	0.215
Non-surgery	164	13 (11.0-15.0)		323	10 (8.4-11.6)	1.09 (0.90-1.33)	0.362
^*^ Municipal and county hospitals *vs* provincial hospitals							

与非手术治疗相比，任意TNM分期手术治疗的肺癌死亡危险性明显下降(*P* < 0.001)(表 5)，其中早期病例手术治疗的生存状况改善幅度大于晚期病例; 腺癌、鳞癌手术治疗的中位生存时间均有明显增加(*P* < 0.001)。而非手术治疗与未治疗病例相比，仅在Ⅲ期显示统计学差异(*P* < 0.001)。

**5:** 按TNM分期、组织类型分层的手术与非手术、未治疗病例生存状况对比 Survival in lung cancer with different treatment status and pathology by stage at diagnosis

Index	*n*	Median survival (mon, 95%CI)	Hazard ratio (95%CI)	*P*
Clinical stage				
Ⅰ & Ⅱ				
Treatment with surgery	62	60 (50.6-69.4)	1.00 (Ref)	
Treatment without surgery	38	24 (1.1-46.9)	2.58 (1.48-4.48)^a^	0.001
Non-treated	53	25 (15.4-34.6)	1.11 (0.70-1.75)^b^	0.651
Ⅲ				
Treatment with surgery	33	30 (10.0-50.0)	1.00 (ref)	
Treatment without surgery	96	15 (12.6-17.4)	2.06 (1.40-3.03)^a^	0.008
Non-treated	130	10 (8.4-11.6)	1.40 (1.06-1.84)^b^	0.011
Ⅳ				
Treatment with surgery	38	12 (7.8-16.2)	1.00 (ref)	
Treatment without surgery	173	7 (5.5-8.5)	1.93 (1.41-2.64)^a^	0.001
Non-treated	160	5 (3.5-6.5)	0.97 (0.77-1.21)^b^	0.774
Missing/Unknown				
Treatment with surgery	82	50 (33.8-66.2)	1.00 (ref)	
Treatment without surgery	180	12 (9.8-14.2)	2.92 (2.24-3.80)^a^	0.000
Non-treated	205	11 (8.6-13.4)	0.92 (0.75-1.14)^b^	0.434
Pathological type				
Adenocarcinoma				
Treatment with surgery	137	40 (30.3-49.7)	1.00 (Ref)	
Treatment without surgery	81	11 (8.6-13.4)	3.01 (2.06-4.40)^a^	0.000
Non-treated	13	9 (3.1-14.9)	1.04 (0.57-1.89)^b^	0.887
Squamous cell				
Treatment with surgery	31	60 (45.8-74.2)	1.00 (ref)	
Treatment without surgery	110	9 (6.5-11.5)	3.41 (2.39-4.86)^a^	0.000
Non-treated	166	10 (8.2-11.8)	0.96 (0.75-1.24)^b^	0.770
^a^The reference is treatment with surgery; ^b^The reference is treatment without surgery.				

表 6显示，各分期鳞癌与腺癌相比，仅在Ⅲ期、未知分期的生存率观察到有统计学差异(*P* < 0.005)，鳞癌的死亡危险性分别增加了75.8%和87.7%;相同治疗方式的腺癌与鳞癌之间的生存状况无统计学差异(*P* > 0.05)。

**6:** TNM分期、治疗方式分层的腺癌、鳞癌病例中位生存状况对比 Survival comparison of between squamous cell carcinoma and adenocarcinoma of the lung by treatment status and stage

	Adenocarcinoma			Squamous cell		Hazard ratio^*^(95%CI)	*P*
	*n*	Median survival (mon, 95%CI)		*n*	Median survival (mon, 95%CI)		
TNM stage							
Ⅰ & Ⅱ	50	57 (44.2-69.8)		21	50 (28.2-71.8)	0.92 (0.5-1.71)	0.789
Ⅲ	59	15 (10.3-19.7)		72	10 (8.7-11.3)	1.76 (1.22-2.54)	0.001
Ⅳ	52	8 (5.7-10.3)		113	9 (6.7-11.3)	1.15 (0.83-1.61)	0.419
Missing/Unknown	70	26 (20.1-31.9)		101	11 (7.4-14.6)	1.88 (1.35-2.6)	0.000
Treatment status							
Surgery	137	40 (30.3-49.7)		31	60 (45.8-74.2)	0.83 (0.52-1.3)	0.167
Non-surgery	81	11 (8.6-13.4)		110	9 (6.5-11.5)	1.14 (0.85-1.54)	0.355
Non-treated	13	9 (3.1-14.9)		166	10 (8.2-11.8)	0.96 (0.75-1.23)	0.988
^*^ Squamous cell *vs* Adenocarcinoma							

### 2.5 Cox多因素回归分析

对TNM分期、组织学资料完整的403例*Cox*比例风险模型多因素回归分析(*Backward LR*法)结果见表 7，治疗方法、TNM分期、治疗医院级别、X线胸片筛查是肺癌生存的独立预后因素，其中TNM分期、治疗方法对生存预后影响较大。与Ⅳ期相比，Ⅰ期病例死亡危险性下降了7.68倍。手术治疗的死亡危险性比非手术治疗、未治疗病例分别降低1.6倍和2.84倍(*P* < 0.001); 与未治疗病例相比，非手术治疗的死亡风险下降了47.5%(HR=0.68, 95%CI: 0.48-0.96, *P* < 0.05)。

**7:** Cox多因素分析结果 Result of Cox regression analysis

	*n*	Hazard ratio (95%CI)	*P*
Age (yr)		1.00 (0.98-1.00)	0.440
Residence areas			
Urban	91		
Rural	312	1.01 (0.76-1.34)	0.943
Occupation			
Non-farmer	26		
Farmer	377	1.21 (0.74-1.98)	0.444
Hospital level of service			
Province	177		
Municipal and county	163	1.37 (1.02-1.83)	0.035
X-ray screen			
Screened	33		
Unscreened	370	1.5 (0.97-2.32)	0.067
Pathological type			
Adenocarcinoma	161		
Squamous	206	0.88 (0.66-1.17)	0.367
Other specified types	36	0.88 (0.57-1.37)	0.578
Clinical stage			
Ⅰ	26		
Ⅱ	54	3.07 (1.46-6.44)	0.003
Ⅲ	145	6.07 (3.00-12.26)	0.000
Ⅳ	178	8.68 (4.30-17.52)	0.000
Treatment			
Surgery	133		
Treatment without surgery	147	2.61 (1.85-3.68)	0.000
Non-treated	123	3.84 (2.51-5.88)	0.000

## 3 讨论

肺癌是死亡率最高的恶性肿瘤，5年生存率长期保持在20%以下^[17]^，2005年-2009年欧美、东亚等发达国家年龄标化5年生存率在15%以上，而非洲、南美洲、南亚等发展中国家则小于10%^[18]^。国内不同地区之间肺癌生存率也存在较大的差距，2006年-2008年中国^[19, 20]^(东部地区为主的17个肿瘤登记点)城市5年ASRS比农村高82.4%(19.7% *vs* 10.8%)，其中女性差距更大，城市居民是农村的1.9倍(22.6% *vs* 11.9%)。宣威市、富源县是西南欠发达农村地区，尚未见生存状况报告，本研究对象占当地同期诊断肺癌的87.6%，研究结果基本上可以代表其女性肺癌人群的生存状况。2006年-2010年诊断的非吸烟女性肺癌早期和手术治疗病例仅占12.2%和17.2%，而未治疗的高达43.8%，中位生存时间13.2个月，5年观察生存率8.9%(RS=9.4%, ASRS=10.1%)，8年观察生存率1.4%(RS=1.7%, ASRS=1.4%)，明显低于国内外发达地区。

TNM分期和手术治疗是影响肺癌生存的主要因素，丹麦^[21]^2002年-2012年人群肺癌随访Ⅰ期患者5年生存率是Ⅳ期12.7倍(38% *vs* 3%)，手术治疗是非手术的7.3倍(44% *vs* 6%); 而肺癌综合治疗相对完善的日本不同分期和治疗方法的生存率(2002年)差别也较大^[22]^，Ia期是Ⅳ期12.2倍(70.5% *vs* 5.8%)，手术治疗是非手术的7.8倍(66.0% *vs* 8.5%)。本次研究对象生存状况与有丹麦、日本不同，其一是生存率较低，而且不同分期间或手术与非手术治疗的差别则更大。调整了其他因素的影响后，Ⅳ期肺癌死亡危险性比Ⅰ期增加7.68倍; 手术治疗的死亡危险性比非手术治疗、未治疗病例分别降低了1.61倍和2.84倍，其优势在不同分期和组织类型均得以体现。单因素分析非手术治疗与未治疗病例相比，生存率无统计学差异(*P* > 0.05); *Cox*分析调整其他因素后非手术治疗的危险性降低了47.5%。医院级别也是影响生存状况的独立预后因素，省级医院治疗的预后明显优于市、县级医院，不完全是因为其早期和手术病例较多，而且其优良的医疗技术资源对Ⅳ期和鳞癌的治疗效果也明显偏优。

肺癌组织类型对生存的影响仍有争议，Strand等^[23]^研究显示鳞癌是一个独立的有利预后因素，SEER^[24]^和WJOG ^[25]^人群监测结果显示，腺癌是晚期肺癌的重要有利预后因素; 也有研究发现^[26]^鳞癌和非鳞癌生存率没有明显差异。本研究单因素分析显示腺癌生存预后优于鳞癌，而TNM分期和组织学类型信息完整病例(占32.4%)的多因素*Cox*分析结果未予支持。

Dominioni^[27]^、Oken^[28]^人群X线胸部筛查可得到较多的早期肺癌病例，提高生存率，但不能降低死亡率^[29]^，本研究中的X线胸部筛查发现病例的生存率有一定提高，主要是因为手术治疗比例提高了56.3%，而并没有发现较多的早期病例。

**1:**
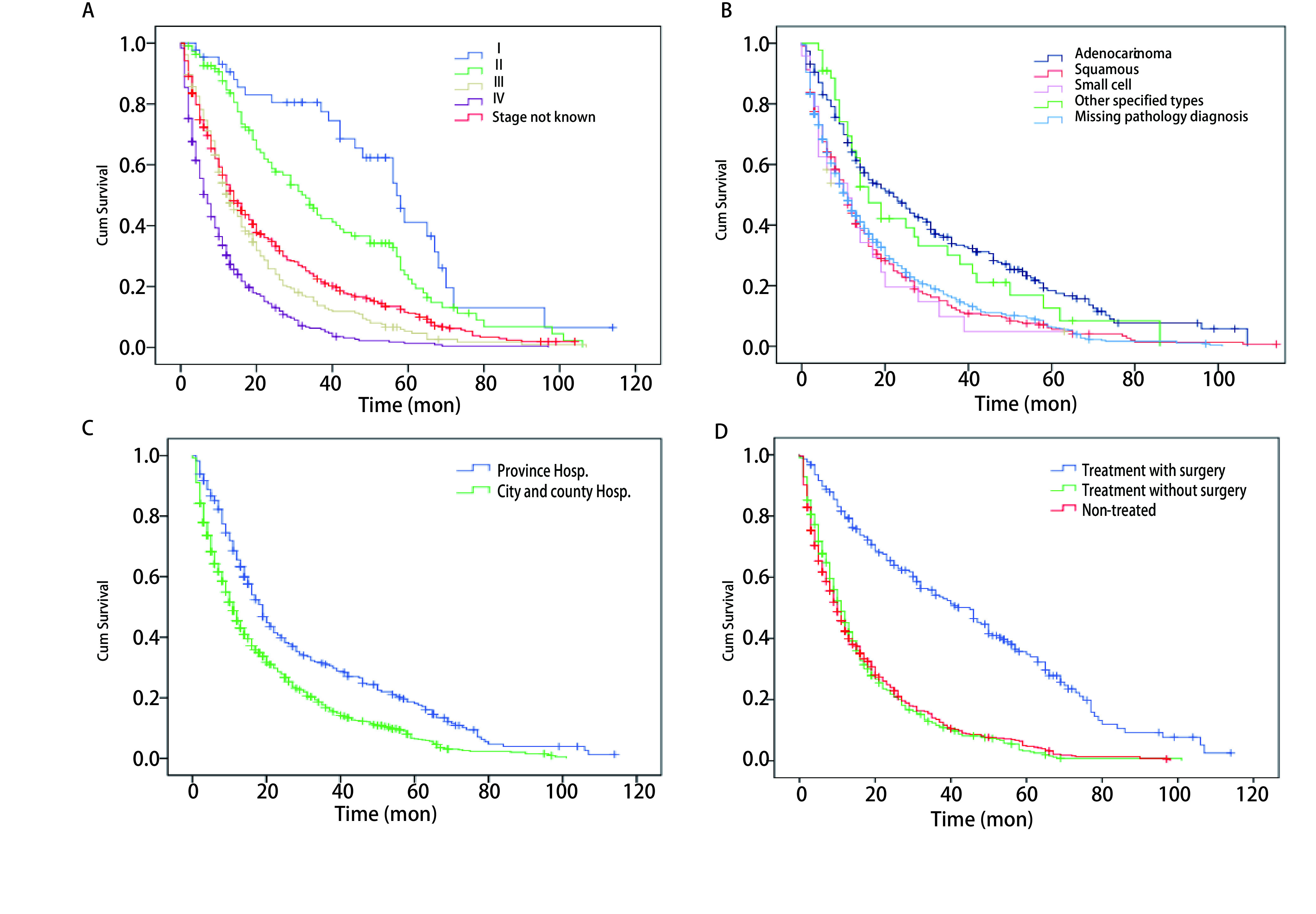
不同临床分期(A)、组织类型(B)、治疗医院级别(C)和治疗方式(D)病例的生存曲线 Kaplan-Meier survival curves for lung cancer by clinical stage (A), subtype (B), hospital level of service (C) and treatment status (D)

地区社会经济水平(收入、文化、社会地位)和居住位置是影响肿瘤生存率的重要因素，较低的社会经济状况导致较差肺癌生存预后^[30, 31]^，甚至可成为早期肺癌的一个独立于手术、种族、婚姻的不良预后因素^[32]^。本研究显示城乡差别、职业不是影响生存的独立预后因素，但不能忽视这些因素的作用。单因素分析发现的城镇居民的生存状况优于乡村居民，主要是他们的经济状况、医疗服务保障或健康意识较好，Ⅰ期、Ⅱ期肺癌和手术比例较高; 而非农民职业生存预后好于农民，其优势仅体现在住院治疗及入住费用高、医疗条件较好的省级医院方面，经济收入、文化水平的优势不一定能够有较强的防癌意识，驱使个人主动筛查，及时发现早期病变。

本文首次报告了滇东农民肺癌高发区的人群肺癌生存状况，研究显示由于社会经济限制(病例诊断时期当地农村居民尚为完全享有医疗保健)和缺乏主动筛查，诊断时早期病例少、未治疗病例多，而手术和规范综合治疗较少等原因致使宣威、富源肺癌患者生存率过低。其次由于组织学诊断、TNM分期信息未完全涵盖全部病例、单因素分析与部分病例*Cox*多因素结果不完全一致等缺陷可能对研究结果产生一定偏倚，有待于进一步补充和完善。

## References

[1] National Office for Cancer Prevention and Control (1980). Investigation of China Cancer Mortality (1973-1975).

[b2] 2He XZ, Yang RD eds. Lung cancer and indoor air pollution from coal burning.1^st^ ed. Kun Ming: Yunnan Science and Technology Press, 1994: 25-76.何兴舟, 杨儒道主编.室内燃煤空气污染与肺癌.第1版.昆明: 云南科技出版社, 1994: 25-76.

[b3] 3National Office for Cancer Research and Control, National Center for Cancer Registry, Disese Control and Prevention Bureau, MOH. China Cancer Mortality Report: the 3^rd^ national retrospective sample survey of the cause of death in China. 1^st^ ed. Beijing: People's Medical Publishing House, 2010: 18-36.全国肿瘤防治研究办公室, 全国肿瘤登记中心, 卫生部疾病预防控制局.中国肿瘤死亡报告: 全国第三次死因回顾抽样调查.第1版.北京: 人民卫生出版社, 2010: 18-36.

[4] Li J, Tang R, Yin G (2004). An investigation on incidence of lung cancer in Fuyuan county, Yunnan, China. Zhongguo Zhong Liu.

[5] Zhi XY, Wu YL, Ma SL (2012). Chinese guidelines on the diagnosis and treatment of primary lung cancer (2011 version). Zhongguo Fei Ai Za Zhi.

[6] Li JH, Zhang YS, Li Y (2011). Descriptive study on the epidemiology of lung cancer in coal-producing area in eastern Yunnan, China. Zhongguo Fei Ai Za Zhi.

[b7] 7Shambaugh EM, Young JU, Zippin C, *et al*. Book7-Statistics and Epidemiology for Cancer Registrars. in: SEER Self Instructional Manuals for Tumor Registrars. Bethesda: NIH Publication, 1994. 115-150

[b8] 8Cho H, Howlader N, Mariotto AB, *et al*. Estimating relative survival for cancer patients from the SEER Program using expected rates based on Ederer Ⅰ versus Ederer Ⅱ method. Available online: https://surveillance.cancer.gov/reports/tech2011.01.pdf

[b9] 9Dickman PW, Hakulien T. Population-based cancer survival analysis. Available online: http://www.pauldickman.com/teaching/tampere2004/book_draft.pdf

[10] Swaminathan R, Brenner H (2011). Stastistical methods for Cancer survival analysis. IARC Sci Publ.

[11] Black RJ, Bashir SA (1998). World standard cancer patient populations: a resource for comparative analysis of survival data. IARC Sci Publ.

[12] Xiang YB, Gao YT, Chen AQ (1995). Application of stratified analysis in survival data. Zhonghua Yu Fang Yu Xue Za Zhi.

[13] Spruance SL, Reid JE, Grace M (2004). Hazard ratio in clinical trials. Antimicrob Agents Chemother.

[b14] 14Definition of the hazard ratio. Available online: http://www.graphpad.com/support/faqid/1226/

[15] Clark TG, Bradburn MJ, Love SB (2003). Survival Analysis Part Ⅰ: Basic concepts and first analyses. Br J Cancer.

[16] Xiang YB (1998). Statistical methods in the analysis of epidemiological data: Risk ratio estimation in single variable analysis of survival data. Zhonghua Liu Xing Bing Xue Za Zhi.

[17] Allemani C, Weir HK, Carreira H (2015). Global surveillance of cancer survival 1995-2009: analysis of individual data for 25, 676 887 patients from 279 population-based registries in 67 countries (CONCORD-2). Lancet.

[18] Allemani C, Matsuda T, Di Carlo V (2018). Global surveillance of trends in cancer survival 2000-14 (CONCORD-3): analysis of individual records for 37, 513, 025 patients diagnosed with one of 18 cancers from 322 population-based registries in 71 countries. Lancet.

[19] Zeng H, Zheng R, Guo Y (2015). Cancer survival in China, 2003-2005: A population-based study. Int J Cancer.

[20] Zeng H, Chen W, Zheng R (2018). Changing cancer survival in China during 2003-15: a pooled analysis of 17 population-based cancer registries. Lancet Glob Health.

[21] Jakobsen E, Rasmussen TR, Green A (2016). Mortality and survival of lung cancer in Denmark: Results from the Danish Lung Cancer Group 2000-2012. Acta Oncol.

[22] Sawabata N, Asamura H, Goya T (2010). Japanese Lung Cancer Registry Study: First prospective enrollment of a large number of surgical and nonsurgical cases in 2002. J Thorac Oncol.

[23] Strand TE, Rostad H, Møller B (2006). Survival after resection for primary lung cancer: a population based study of 3, 211 resected patients. Thorax.

[24] Cetin K, Ettinger DS, Hei YJ (2011). Survival by histologic subtype in stage Ⅳ nonsmall cell lung cancer based on data from the Surveillance, Epidemiology and End Results Program. Clin Epidemiol.

[25] Kogure Y, Ando M, Saka H (2013). Histology and smoking status predict survival of patients with advanced non-small-cell lung cancer: Results of West Japan Oncology Group (WJOG) Study 3906L. J Thorac Oncol.

[26] Lopez Guerra JL, Gomez DR, Lin SH (2013). Risk factors for local and regional recurrence in patients with resected N0-N1 non-small-cell lung cancer, with implications for patient selection for adjuvant radiation therapy. Ann Oncol.

[27] Dominioni L, Rotolo N, Mantovani W (2012). A population-based cohort study of chest X-ray screening in smokers: lung cancer detection findings and follow-up. BMC Cancer.

[28] Oken MM, Marcus PM, Hu P (2005). Baseline chest radiograph for lung cancer detection in the randomized prostate, lung, colorectal and ovarian cancer screening trial. J Natl Cancer Inst.

[29] Gavelli G, Giampalma E (2000). Sensitivity and specificity of chest X-ray screening for lung cancer. Cancer.

[30] Atkins GT, Kim T, Munson J (2017). Residence in rural areas of the United States and lung cancer mortality. disease incidence, treatment disparities, and stage-specific survival. Ann Am Thorac Soc.

[31] Booth CM, Li G, Zhang-Salomons J (2010). The impact of socioeconomic status on stage of cancer at diagnosis and survival: a population-based study in Ontario, Canada. Cancer.

[32] Ou SH, Zell JA, Ziogas A (2008). Low socioeconomic status is a poor prognostic factor for survival in stage Ⅰ nonsmall cell lung cancer and is independent of surgical treatment, race, and marital status. Cancer.

